# Prevalence of occupational accidents and associated variables in the Brazilian workforce

**DOI:** 10.47626/1679-4435-2020-578

**Published:** 2021-03-03

**Authors:** Ana Clara Dantas de Souza, Isabelle Ribeiro Barbosa, Dyego Leandro Bezerra de Souza

**Affiliations:** Programa de Pós-Graduação em Saúde Coletiva, Universidade Federal do Rio Grande do Norte, Natal, RN, Brazil

**Keywords:** occupational health, absenteeism/presenteeism, occupational accident

## Abstract

**Introduction:**

Current estimates suggest that 317 million occupational accidents occur annually worldwide.

**Objectives:**

To estimate the prevalence of occupational accidents and associated variables in the Brazilian workforce.

**Methods:**

A cross-sectional study was performed using data from adults aged 18 or older who participated in the National Health Survey (Pesquisa Nacional de Saúde) (2013). This study was based on participants’ responses to questions regarding their history of occupational accidents in the previous 12 months. Socioeconomic, lifestyle and health-related variables were also examined. Prevalence rates and ratios were calculated using Poisson multivariate regression models (with 95% confidence intervals), followed by Wald’s tests for robust variance estimation.

**Results:**

The prevalence of occupational accidents was 2.79% (95% confidence interval, 2.53-3.08%). These incidents were associated with male gender (prevalence ratio = 1.42; 95% confidence interval, 1.14-1.77), living in rural areas (prevalence ratio = 1.27; 95% confidence interval, 1.06-1.62), age 18 to 24 (prevalence ratio = 2.02; 95% confidence interval, 1.20-3.40), illiteracy (prevalence ratio = 3.12; 95% confidence interval, 1.96-4.96) and having two or more chronic illnesses (prevalence ratio = 2.12; 95% confidence interval, 1.29-3.47).

**Conclusions:**

Though the prevalence of occupational accidents in the Brazilian workforce was low, these incidents were associated with multimorbidity, socioeconomic status and lifestyle variables.

## INTRODUCTION

The study of health and disease processes in the field of occupational health seeks to examine the impact of work on human health and identify the main determinants of disability in the working population. This area of public health focuses on health promotion and protection in this target group, striving to reduce occupational accidents, illnesses and injuries; improve working conditions; and increase workers’ quality of life.^1^

The aging of the workforce and the resulting increase in chronic illnesses have changed the character of occupational health.^2^ Furthermore, new work practices have had a direct influence on workers’ health as a result of increased demands and expectations, greater diversity, and the precariousness of working conditions and social protections, in addition to decreased autonomy, environmental degradation and worsening quality of life.^3^

The frequency of occupational accidents has increased steadily every year. An estimated 317 million such incidents occur every year worldwide.^4^ The International Labor Organization (ILO) also notes that 2.3 million workers every year die from work-related activities.^5^

The latest report of the 4th National Conference on Occupational Health notes that 717,911 occupational accidents were reported in Brazil in 2013, and that these incidents were among the main causes of absenteeism in the country.^6^ The fact that 18.7 million Brazilians work in the informal sector and have no access to social or labor protections^7^ is a major reason why health and safety incidents go unreported.

In Brazil, the National Workers’ Health Policy issued by the Ministry of Health is responsible for promoting the reduction of occupational accidents and illnesses through the National Network of Occupational Health Care (Renast), composed of State and Regional Reference Centers for Workers’ Health (Cerest), which provide the technical and scientific basis for the improvement of working conditions and worker quality of life.^3^

Occupational accidents are preventable^8^ and their continued occurrence reflects the fragility of programs and policies for health promotion and disease prevention in workers,^9^ including administrative strategies aimed at protecting workers’ health and preventing occupational accidents such as regulations on the use of personal (PPE) and collective protective equipment (CPE). The literature estimates that countries dedicate 4 to 10% of their gross domestic product (GDP) to the management of occupational illnesses, noting that this figure may be even higher developed or developing countries such as Brazil.^10^

Therefore, it is important to study the epidemiology of occupational accidents, assessing their prevalence and determinants in the Brazilian population. Additionally, by analyzing the characteristics of the most severely affected individuals, strategic actions can be implemented in the field of occupational health surveillance, contributing to the development of protocols that meet the needs of Brazilian workers, allowing for effective prevention and control measures in both formal and informal work settings.

The aim of this study was to estimate the prevalence of occupational accidents in Brazilian workers and identify the socioeconomic, lifestyle and health variables associated with these incidents.

## METHODS

An association study was performed using secondary data from the 2013 National Health Survey (PNS) in Brazil. The PNS is a national household survey designed to characterize and assess the Brazilian population in terms of their health status, lifestyle, chronic illnesses, risk factors, as well as health care access and utilization.^11^

The population sampled by the PNS consists of adults aged 18 years or older, living in private households throughout the country.^12^ The sample was stratified by three levels, with census tracts as the primary sampling or collection units; households as secondary units; and adult residents (≥18 years) as the tertiary sampling units. The final sample consisted of 64,308 individuals, with a response rate of 86%.^13^ The present study focused on respondents who were employed at the time of the survey, resulting in a sub-sample of 47,629 participants.

Data were extracted from Module O of the PNS, entitled “Accidents and Violence,” and the dependent variable was defined as an affirmative response to the following question: O21. In the past 12 months, have you been involved in an occupational accident (excluding traffic accidents)?

The dependent variable was operationalized based on the definition of occupational accidents provided in article 19 of law 8.213/1991,^14^ which states that,

An occupational accident is an event that occurs in the course of employment or as a result of occupational activities [...] resulting in physical harm or functional impairment leading to death or the temporary or permanent loss or reduction in occupational functioning [free translation].

The independent variables considered in this study were: age group (18-24 years; 25-39 years; 40-59 years; 60 years or older); education level (illiterate [no education or incomplete primary school]; primary [complete primary and incomplete secondary school]; secondary [complete secondary school and incomplete university]; or university [undergraduate degree]); marital status (living with partner; not living with partner); place of residence (urban or rural); smoking status (never; current smoker; former smoker); alcohol intake (none; moderate; excessive); private medical or dental insurance (yes or no); socioeconomic status (classes A and B; class C; classes D and E); multimorbidity (0; 2; 3; 4); and number of weekly hours (the only quantitative variable in the study, used to classify participants according to weekly working hours).

The prevalence of occupational accidents in each category of the socioeconomic and health variables studied was calculated using statistical analysis. Bivariate analyses were used to calculate prevalence ratios (PR) and 95% confidence intervals (95%CI) at p < 0.05. Variables with significant results in the bivariate analysis (p < 0.2) were entered as predictors in a multivariate Poisson regression followed by Wald’s tests for robust variance estimation. Sample weights were based on the full sample of the PNS. Analyses were performed using Stata, version 14.0.

The PNS was approved by the National Research Ethics Committee (CONEP) on July 8, 2013, under protocol number 10853812.7.0000.0008. The present study was based on secondary data from the PNS collected from the official website of the Ministry of Health, and does not require ethical approval, as outlined in the National Health Council Resolution 466/2012.

## RESULTS

The prevalence of occupational accidents was 2.79% (95%CI, 2.53-3.08). Descriptive analyses showed that individuals who consumed moderate or excessive amounts of alcohol were the most likely to have experienced an occupational accident in the past 12 months. Other common characteristics in individuals with a history of occupational accidents included male gender, being a current or former smoker, having a low education level, two or more chronic illnesses, living in a rural area, having no health insurance, having a low socioeconomic position and being in the 25- to 39-year age group (Table 1).

**Table 1:** Twelve-month prevalence (%) of occupational accidents in the Brazilian working population, according to socioeconomic and health characteristics assessed by the National Health Survey (PNS), 2013 (n = 47,629)

Variables	P (%)	95%CI	
		LL	UL
Occupational accident			
No	97.21	96.92	97.47
Yes	2.79	2.53	3.08
Gender			
Male	3.59	3.17	4.06
Female	1.92	1.63	2.26
Age (years)			
18-24	3.19	2.50	4.08
25-39	2.98	2.58	3.44
40-59	2.80	2.37	3.30
60 and older	1.61	1.10	2.35
Marital status			
No	2.61	2.19	3.11
Yes	2.90	2.58	3.26
Alcohol intake			
None	2.67	2.37	3.01
Moderate	2.63	2.10	3.30
Excessive	4.38	3.40	5.62
Smoking status			
Nonsmoker	2.41	2.11	2.76
Current smoker	3.58	2.97	4.33
Former smoker	3.64	2.94	4.49
Education level			
Illiterate	3.18	2.25	4.47
Primary	3.40	2.94	3.93
Secondary	3.01	2.55	3.55
Higher education	1.30	1.01	1.67
Place of residence			
Urban	2.63	2.35	2.93
Rural	3.87	3.14	4.77
Medical or dental insurance			
No	3.15	2.82	3.52
Yes	1.99	1.64	2.43
Multimorbidity			
0	2.63	2.34	2.94
2	3.39	2.57	4.47
3	3.68	2.56	5.25
4	3.32	2.18	5.03
Socioeconomic status			
A and B	2.44	1.93	3.08
C	2.86	2.45	3.34
D and E	2.97	2.59	3.42

95%CI = 95% confidence interval; LL = lower limit; P = prevalence; UL = upper limit.

Table 2 shows the results of bivariate associations between occupational accidents and socioeconomic, health-related and occupational characteristics. Occupational accidents were significantly associated with male gender (PR = 1.87; 95%CI, 1.52-2.29), lack of health insurance (PR = 1.58; 95%CI, 1.26-1,97), illiteracy (PR = 2.43; 95%CI, 1.58-3.74), primary education (PR = 2.61; 95%CI, 1.96-3.46), secondary education (PR = 2.31; 95%CI, 1.71-1.11), living in rural areas (PR = 1.47; 95%CI, 1.16-1.86), being in a younger age group and excess alcohol consumption (PR = 1.63; 95%CI, 1.25-2.15). Multimorbidity was significantly associated with occupational accidents at p = 0.12, and was therefore included in the multivariate model.

**Table 2:** Bivariate analysis of factors related to experiencing occupational accidents in the past 12 months in the Brazilian working population, according to the National Health Survey (PNS), 2013 (n = 47,629)

Variables	PR	95%CI		p-value
		LL	UL	
Gender				< 0.01
Male	1.87	1.52	2.29	
Female	1.00			
Age (years)				< 0.01
18-24	1.98	1.28	3.07	
25-39	1.85	1.23	2.78	
40-59	1.74	1.15	2.63	
60 and older	1.00			
Marital status				0.32
No	1.00			
Yes	1.10	0.90	1.36	
Medical or dental insurance				< 0.01
No	1.58	1.26	1.97	
Yes	1.00			
Alcohol intake				< 0.01
None	1.00			
Moderate	0.98	0.76	1.27	
Excessive	1.63	1.24	2.15	
Smoking status				0.64
Nonsmoker	1.00			
Current smoker	0.91	0.49	1.69	
Former smoker	1.22	0.76	1.94	
Education level				< 0.01
Illiterate	2.43	1.58	3.74	
Primary	2.61	1.96	3.46	
Secondary	2.31	1.71	1.11	
Higher education	1.00			
Place of residence				< 0.01
Urban	1.00			
Rural	1.47	1.16	1.86	
Multimorbidity				0.12
0	1.00			
2	1.29	0.95	1.74	
3	1.40	0.96	2.03	
4	1.26	0.81	1.95	
Socioeconomic status				0.34
A and B	1.00			
C	1.21	0.80	1.82	
D and E	0.91	0.62	1.33	
Weekly working hours	1.00	1.00	1.01	< 0.01

95%CI = 95% confidence interval; LL = lower limit; p-value = probability of significance; PR = prevalence ratio; UL = upper limit.

The multivariate analysis in Table 3 showed that the following variables were still associated with occupational accidents in the final model: 18-24 years of age (PR = 2.02; 95%CI, 1.20-3.40), illiteracy (PR = 3.12; 95%CI, 1.96-4.96), two or more chronic illnesses (PR = 2.12; 95%CI, 1.29-3.47), male gender (PR = 1.42; 95%CI, 1.14-1.77) and living in a rural area (PR = 1.27; 95%CI, 1.06-1.62).

**Table 3:** Multivariate-adjusted prevalence ratios (PRadj) for occupational accidents in the past 12 months in the Brazilian working population, based on the National Health Survey (PNS), 2013

Variables	PRadj	95%CI		p-value
		LL	UL	
Gender				< 0.01
Male	1.42	1.14	1.77	
Female	1.00			
Age (years)				
18-24	2.02	1.20	3.40	
25-39	1.79	1.11	2.88	
40-59	1.41	0.87	2.28	
60 and older	1.00			
Education level				< 0.01
Illiterate	3.12	1.96	4.96	
Primary	2.76	2.02	3.78	
Secondary	2.32	1.70	3.19	
Higher education	1.00			
Multimorbidity				< 0.01
0	1.00			
2	1.70	1.22	2.36	
3	1.90	1.27	2.84	
4	2.12	1.29	3.47	
Place of residence				< 0.04
Urban	1.00			
Rural	1.27	1.00	1.62	
Weekly working hours	1.00	0.99	1.01	0.07

95%CI = 95% confidence interval; LL = lower limit; P = prevalence; p-value = probability of significance; UL = upper limit.

## DISCUSSION

The present findings showed that the prevalence of occupational accidents in the 12 months prior to the PNS was lower than that reported in previous studies in Brazil. A study by Oliveira and Paiva^15^ involving public service workers in a mobile emergency service across four cities in the state of Minas Gerais observed a prevalence rate of 29.40% for occupational accidents in this sample. In a study of tobacco farmers in the city of São Lourenço do Sul, state of Rio Grande do Sul, Brazil, Zago et al.^16^ found a prevalence of occupational accidents to be 4.5%.

It is important to note that the present findings refer to the overall prevalence of occupational accidents in the country rather than a single occupational category, like the previously cited studies. Though this study could not determine the occupations in which accidents were most frequent, the use of a population-based survey allowed us to quantify the magnitude of this issue among Brazilian workers with a higher degree of robustness.

According to the Annual Statistical Reports of the Social Security Administration (AEPS), a total of 737,378 occupational accidents were reported in 2013.^17^ Yet if the prevalence rates observed in the present study for the year prior to the PNS were applied to the working population of Brazil, which was 86,353,839 in 2010,^18^ we would obtain a figure of 2,409,272 occupational accidents. These results confirm the high number of accidents in Brazil, which also has the highest global percentage of occupational accidents leading to death.^17^ The present findings also suggest that only a third of these incidents are accounted for in official statistics. This may correspond to the share of accident victims covered by social insurance.

Population-based studies that describe the occurrence of occupational accidents are especially important, since in Brazil, the underreporting of illnesses and injuries prevents an accurate estimation of the impact of work on the health of the working population, especially for those in the informal sector, which constitutes a major limitation of occupational health surveillance in Brazil.^19^

The need for accurate information on the profile of workers and the frequency of occupational illness and injury led to the passing of Ordinance No. 777/GM on 28 April 2004, which “describes the technical procedures for the mandatory reporting of occupational health issues to a sentinel surveillance network within the Unified Health System -SUS,” and requires that all occupational and work-related health issues be reported to the Information System on Notifiable Diseases (Sinan).^20^ This system is crucial for the surveillance and monitoring of major occupational health issues, and as such, provides a basis for the development of strategies and initiatives to promote and protect occupational health.

It is estimated that 9% of wages and 60% of benefits paid by Social Security in Brazil (2013) are associated with special retirement payments or compensation for work-related illnesses, injuries and accidents;^21^ this figure amounts to 71 billion reais per year.^22^

Therefore, in an attempt to encourage companies to take better care of workers and reduce the frequency of occupational accidents, a 2007 decree by the Ministry of Social Security determined that companies should reserve a percentage of payroll ranging from 1 to 3% to cover social security benefits associated with work-related accidents.^23^ Additionally, 2018 saw the institution of the Accident Prevention Factor (FAP), which from 2019 onward was used to guarantee Work Accident Insurance (SAT) and encourage companies with lower accident rates to contribute only 1% of payroll to compensation payments, rather than the 3% required for companies with a higher frequency of occupational accidents and illnesses.^24^

The costs of occupational accidents to the SUS can be calculated based on Hospital Admission Authorization (AIH) records. These data suggest that R$ 422,409.43 were used to cover expenses related to occupational accidents between 2010 and 2014.^25^ According to scientific evidence, the SUS is a major “provider of funds and assistance to victims of occupational accidents and illnesses,” accounting for over 50% of the services provided to these individuals while private and philanthropic institutions contribute a smaller share (nearly 40.3%), underscoring the financial impact of these incidents on the SUS.^26^

In addition to the financial burden associated with occupational accidents, it is important to consider the multidimensional social impact of these incidents on the victims and their families, who will have to face death, loss or reduction in occupational functioning as a result of these events. In 2009, nearly 16 billion reais were paid by workers and their families in compensation for work injuries.^22^

People with multimorbidity also reported a higher frequency of occupational accidents. Frey et al.^27^ noted that individuals with four or more chronic illnesses were 2.35 times more likely to experience work accidents than individuals with two or three conditions. Multimorbidity may lead to fragility, especially when at least one of the illnesses present is unstable. According to a systematic review of longitudinal studies on multimorbidity, the literature has reached a consensus on the role of mental disorders as some of the most significant and incapacitating illnesses in individuals with two or more chronic conditions. As such, he presence of multiple chronic illnesses, be they physical or mental, is directly linked to absenteeism and decreased productivity, increasing the financial burden on the system.^28^

Male gender was also strongly associated with the occurrence of occupational accidents. According to the Brazilian Institute of Geography and Statistics (IBGE), men (3.5 million) aged 18 to 24 years were twice as likely as women (1.5 million) to experience occupational accidents.^12^ Additionally, in an epidemiological study of male mortality rates in Brazil, Oliveira et al.^9^ found that men aged 20 to 59 years accounted for 35.2% of deaths due to external causes in the year 2010.^29^ The IBGE reports that males constituted 56.7% of the Brazilian population in 2013, while women comprised the remaining 43.3%;^12^ as such, the previously mentioned issues reflect the predominantly male workforce.

Individuals aged 18 to 24 were especially likely to report occupational accidents, and did so at similar rates to those seen in previous studies, where the 18- to 39-year age group is found to be most affected by these incidents. In a study of secondary data from accident reporting systems, Gonçalves et al.^4^ found that 62.5% of occupational accidents affected workers aged 18 to 29 years. This may be due to their socioeconomic status and the frequency with which they hold low-qualification jobs, leading to increased exposure to dangerous situations with a higher accident risk.^6^

The analysis of education levels revealed that individuals with no education (illiterate) were three times more likely to have experienced an occupational accident than those with higher education levels. This may be due to a lack of access to information and health care services, which result in unfamiliarity with basic principles of protection and prevention, as well as health promotion initiatives.^29^ Similarly, in a study of secondary data from several information sources, Hennington and Monteiro^30^ corroborated the present findings, nothing that individuals with lower education levels (35.5%) are most affected by occupational accidents.

Living in rural areas was also associated with a higher frequency of occupational accidents. This may be due to difficulty accessing health care services, a lack of resources and transportation to travel to and from work, as well as the level of commitment to labor laws.^29^ On the other hand, Hennington and Monteiro^30^ found that more accidents happen in urban settings (90.2%), and suggested that this may be due to the increased access to health care associated with living in urban areas, which results in a higher reporting rate for occupational accidents.

The cross-sectional designed used in the present study prevented the identification of causal relationships between variables, since the direction of causality cannot be determined when risk factors and outcomes are assessed simultaneously. This constitutes a limitation of the present study. A second potential limitation is that, although the present study was based on a representative sample of the population, diagnoses were self-reported. As a result, findings may have been influenced by comprehension issues, recall bias and under- or overestimated prevalence rates. However, the sample size and methodological rigor add to the robustness of the present findings. Since this was a population-based study, it also contributes to the identification of outcomes and risk factors across the country.

## CONCLUSIONS

This study revealed a low prevalence of occupational accidents in Brazilian workers. This finding may have been influenced by the recall bias of respondents or a lack of understanding of the multidimensional nature of occupational accidents. The present findings differ from official global statistics, according to which Brazil ranks fourth in occupational accidents, behind China, the United States and Russia, respectively.^17^

The lack of questions regarding the nature of each participants’ occupation and the types of accident or injury they experienced may also have affected the quality of responses. Future population-based studies in Brazil, such as the PNS, should collect additional information on the frequency and consequences of occupational accidents, in order to address the limitations identified in the present study and provide a more accurate view of the issues facing Brazilian workers. There is also a need for longitudinal population -based studies that analyze the association between multimorbidity and work-related problems, which could not be assessed in the present study, but remains a topic of interest in the international literature on worker health.^28^

Despite these limitations, the present study makes a significant contribution to the literature by identifying variables that influence the occurrence of workplace accidents. These findings can support and encourage strategic actions and policies to monitor the state of occupational health and promote a discussion of the importance of reporting all occupational accidents, injuries and illnesses, creating a culture of reporting in both public and private services in Brazil.

## References

[1] Mendes EA, Teixeira LR, Bonfatti RJ (2017). As condições de saúde dos trabalhadores a partir dos exames periódicos de saúde. Saúde Debate.

[2] Organização Pan-Americana da Saúde (2019). Saúde do Trabalhador.

[3] Pinheiro TMM, Dias EC, Silveira AM, Silva JM (2014). Saúde do trabalhador.

[4] Gonçalves SBB, Sakae TM, Magajewski FL (2018). Prevalência e fatores associados aos acidentes de trabalho em uma indústria metalomecânica. Rev Bras Med Trab.

[5] Batista J, Rodrigues SC, Lordani TVA, Andolhe R (2015). Caracterização de vítimas de acidentes laborais atendidas em unidades de pronto atendimento da região Sul/Brasil. Rev Enferm UFSM.

[6] Brasil, Ministério da Saúde (2015). 4ª Conferência Nacional de Saúde do Trabalhador e da Trabalhadora. Relatório Final.

[7] Almeida GCM, Medeiros FCD, Pinto LO, Moura JMBO, Lima KC (2016). Prevalência e fatores associados e acidentes de trânsito com mototaxistas. Rev Bras Enferm.

[8] Santana VS, Araújo-Filho JB, Albuquerque-Oliveira PR, Barbosa-Branco A (2006). Acidentes de trabalho: custos previdenciários e dias de trabalho perdidos. Rev Saude Publica.

[9] Oliveira LG, Almeida CVD, Barroso LP, Gouvea MJC, Muñoz DR, Leyton V (2016). Acidentes de trânsito envolvendo motoristas de caminhão no Estado de São Paulo: prevalência e preditores. Cienc Saude Colet.

[10] Sá ACMGN, Gomide MHM, Sá ATN (2017). Acidentes de trabalho suas repercussões legais, impactos previdenciários e importância da gestão no controle e prevenção: revisão sistemática de literatura. Rev Med Minas Gerais.

[11] Souza-Júnior PRB, Freitas MPS, Antonaci GA, Szwarcwald CL (2015). Desenho da amostra da Pesquisa Nacional de Saúde 2013. Epidemiol Serv Saude.

[12] Brasil, Instituto Brasileiro de Geografia e Estatística (2013). Pesquisa Nacional de Saúde 2013: Acesso e utilização dos serviços de saúde, acidentes e violências.

[13] Damacena GN, Szwarcwald CL, Malta DC, Souza-Júnior PRB, Vieira MLFP, Pereira CA (2015). O processo de desenvolvimento da Pesquisa Nacional de Saúde no Brasil, 2013. Epidemiol Serv Saude.

[14] Brasil, Presidência da República (1991). Lei n. 8.213, de 24 de Julho de 1991. Dispõe sobre os Planos de Benefícios da Previdência Social e dá outras providências.

[15] Oliveira AC, Paiva MHRS (2013). Análise dos acidentes ocupacionais com material biológico entre profissionais em serviços de atendimento pré-hospitalar. Rev Latino-Am Enfermagem.

[16] Zago AM, Meucci RD, Fiori N, Carret MLV, Faria NMX, Fassa AG (2018). Prevalência e fatores associados à acidente de trabalho em fumicultores do município de São Lourenço do Sul, RS, Brasil. Ciênc Saúde Colet.

[17] Pinto JM (2017). Tendência a incidência de acidentes e doenças de trabalho no Brasil: aplicação do filtro Hodrick-Prescott. Rev Bras Saúde Ocup.

[18] Cardoso MCB, Araújo TM (2016). Os Centros de Referências em Saúde do Trabalhador e as ações em saúde mental: um inquérito no Brasil. Rev Bras Saude Ocup.

[19] Cavalcante CAA, Cossi MS, Costa RRO, Medeiros SM, Menezes RMP (2015). Análise crítica dos acidentes de trabalho no Brasil. Rev Atenção Saude.

[20] Almeida PCA, Barbosa-Branco A (2011). Acidentes de trabalho no Brasil: prevalência, duração e despesa previdenciária dos auxílios-doença. Rev Bras Saude Ocup..

[21] Malta DC, Stopa SR, Silva MMA, Szwarcwald CL, Franco MS, Santos FV (2017). Acidente de trabalho autorreferidos pela população adulta brasileira, segundo dados da Pesquisa Nacional de Saúde, 2013. Cienc Saude Colet.

[22] Duarte S (2016). O impacto financeiro e social dos acidentes de trabalho e o processo de adoecimento na indústria frigorífica.

[23] Soares LJP (2008). Os impactos financeiros dos acidentes do trabalho no orçamento brasileiro: uma alternativa política e pedagógica para redução dos gastos.

[24] Brasil, Previdência Social (2018). FAP - Fator Acidentário de Prevenção. Site do Ministério da Economia.

[25] Machado JMH (2014). Impactos Sociais sobre a Folha da Previdência Social e sobre as Despesas do SUS com os Acidentes de Trabalho no Brasil e na Bahia. Relatório do CGST/DSAST/SVS/MS.

[26] Santana VS, Araújo GR, Espírito-Santo JS, Araújo-Filho JB, Iriart J (2007). A utilização de serviços de saúde por acidentados de trabalho. Rev Bras Saude Ocup..

[27] Frey JJ, Osteen PJ, Berglund PA, Jinnett K, Ko J (2015). Predicting the impact of chronic health conditions on workplace productivity and accidents: results from two US Department of Energy national laboratories. J Occup Environ Med.

[28] Cabral GG, Souza ACD, Barbosa IR, Jerez-Roig J, Souza DLB (2019). Multimorbidity and its impact on workers: a review of longitudinal studies. Saf Health Work.

[29] Rios MA, Nery AA, Rios PAA, Casotti CA, Cardoso JP (2015). Fatores associados a acidentes de trabalho envolvendo trabalhadores informais do comércio. Cad Saude Publica.

[30] Hennington EA, Monteiro M (2006). O perfil epidemiológico dos acidentes de trabalho no Vale dos Sinos e o sistema de vigilância em saúde do trabalhador. Hist cienc saude-Manguinhos.

